# Drivers of COVID-19 infections: Perspectives of managers in the Gauteng Department of Health, South Africa

**DOI:** 10.4102/safp.v67i1.6107

**Published:** 2025-09-20

**Authors:** Cyril B. Fonka, Zainonisa Petersen, Nicola Christofides

**Affiliations:** 1 School of Public Health, Faculty of Health Sciences, University of the Witwatersrand, Johannesburg, South Africa; 2 Public Health, Societies and Belonging Division, Human Sciences Research Council, Durban, South Africa

**Keywords:** COVID-19, infections, Gauteng province, health managers, perceptions, factors

## Abstract

**Background:**

This study investigated the perceptions and experiences of Gauteng Department of Health (GDoH) senior managers about factors that contributed to the high incidence of coronavirus disease 2019 (COVID-19) in Gauteng, the hardest hit province in South Africa.

**Methods:**

An exploratory qualitative study was conducted using online in-depth interviews with senior managers in Gauteng. Audio recordings were transcribed verbatim, coded and thematically analysed in NVivo 10. Data saturation was reached at 13 participants (*n* = 13). Findings were reported in line with the Consolidated Criteria for Reporting Qualitative Research (COREQ).

**Results:**

Two main themes emerged from the analysis. Theme I: Perceptions of the burden of COVID-19 in Gauteng. Theme II: Key health and behavioural factors, including poor protocol adherence, exacerbated the spread of COVID-19. Economic challenges such as limited employment prospects and informal settlements, sociocultural enablers like vaccine hesitancy, social media misinformation, limited vaccine and treatment options, and environmental factors such as the OR Tambo International Airport contributed to high population density and heightened the infections and transmission of COVID-19. Governance issues, including corruption in personal protective equipment procurement and embezzlement of COVID-19 funds, undermined the GDoH response.

**Conclusion:**

Understanding perceptions of factors that influence disease transmission is crucial for effectively managing infectious diseases like COVID-19 and future outbreaks. Addressing infrastructure gaps in underserved communities and strengthening government regulations could help to reduce congestion in Gauteng, ultimately reducing the spread of contagious diseases.

**Contribution:**

The study presents a model for investigating and addressing the human factors that drive the transmission of infectious diseases.

## Introduction

Coronavirus disease 2019 (COVID-19) is an infectious disease caused by SARS-CoV-2 and was declared a global pandemic by the World Health Organization (WHO) on 11th March 2020.^1^ By March 2023, over 758 million confirmed cases and 6.8 million COVID-19 deaths were reported worldwide.^2^ The burden of COVID-19 varies across the world. Europe bore the highest burden (36%) of global infections while Africa contributed the lowest at 1.3%. Due to their large populations, United States (US), China and India reported the highest number of COVID-19 infections, respectively.^2^

The explanations for the incidence rate of COVID-19 in any setting include biological or clinical, environmental or natural and human factors. Clinical evidence suggests that individuals with underlying medical conditions, chronic diseases, or compromised immunity due to comorbidities (such as those with HIV and TB, the elderly, pregnant women, and children) were disproportionately affected. The Burden was especially pronounced in low- amd middle-income countries (LMICs) where health systems were already strained prior to the pandemic.^3^ Additionally, strong evidence from systematic reviews has shown that globally, densely populated areas had a positive statistically significant correlation with COVID-19 infections.^3,4^ Commercial cities and major international airports were equally identified as risk zones for COVID-19 in the US, Italy, Spain and China, which were endemic countries.^5,6^ Also, social determinants of health including poor hygiene and sanitation, and economic deprivation manifested in extreme poverty exacerbated COVID-19 infections.^3,4^ For example, black ethnic minorities in the US with low socioeconomic status were disproportionately affected by COVID-19 compared to their white counterparts.^7^

In countries like Belgium, the United Kingdom, and Egypt, the shortage of personal protective equipment (PPE) increased the vulnerability of healthcare workers, including physicians and nurses, who were at the forefront of the pandemic response. Consequently, many were infected, and some lost their lives.^5^ Moreover, a multitude of studies concluded that vaccine hesitancy, particularly in an era of social media platforms such as X (formerly Twitter), Facebook, YouTube, propagated misinformation and eroded public trust in COVID-19 treatments and vaccines. This contributed to anti-vaccination sentiments, conspiracy theories, vaccine-specific concerns, and altered healthcare-seeking behaviours, all of which adversely influenced viral transmission.^8^ Although there are individual rights and civil liberties, it became concerning when healthcare workers themselves resisted COVID-19 vaccinations, increasing their vulnerability and transmission risk.^9,10^ Therefore, pre-existing medical, demographic, environmental, and social factors as well as inequalities and the influence of social media, contributed to the high incidence and prevalence of COVID-19 globally.^11^

Although Africa was spared from COVID-19 expected mortality, the continent still faced significant impacts,^12^ with South Africa being the hardest hit, experiencing the highest morbidity and mortality rates that peaked with the emergence of the Omicron variant.^13^ The rapid spread of COVID-19 in the country could be seen by comparing the daily mean of 280 COVID-19 cases in Gauteng province a week before the Omicron variant was detected, to a daily average of 800 cases a week after the isolation of Omicron.^14^

During the early phase of the outbreak, in the absence of treatment and vaccines, several non-pharmaceutical interventions were adopted as preventive measures worldwide. South Africa also implemented similar measures including physical distancing, hand sanitisation, wearing of face masks, screening and testing, contact tracing, quarantine and movement restriction through lockdowns and curfews. Interestingly, these interventions were effective in mitigating the transmission of the pandemic, but their socioeconomic consequences were critical.^15^ The strict application of non-pharmaceutical interventions was eased with the rollout of the COVID-19 vaccines. Although vaccines are the most effective public health interventions against infectious diseases, there were several challenges with the rollout. For instance, LMICs lacked the financial viability to purchase COVID-19 vaccines as well as the expertise to manufacture the vaccines.^16^ As discussed earlier, social media also has a role in advancing vaccine hesitancy.

**FIGURE 1:**
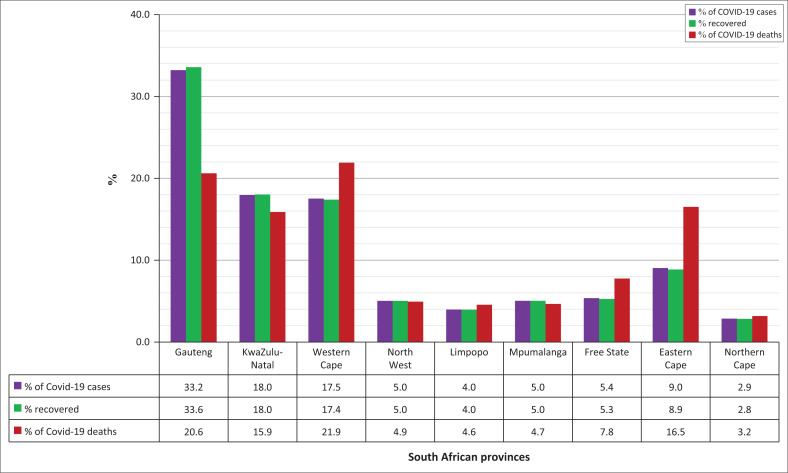
COVID-19 cases, recoveries and deaths in South African provinces (February 2023).

Figure 1 shows that Gauteng province had the highest percentage of reported cases, while Western Cape had the highest percentage of deaths from COVID-19 in South Africa.^17^

The data for Figure 1 was obtained from the South African Coronavirus online portal,^17^ which highlights the high incidence of COVID-19 in Gauteng province. Although demographic, socioeconomic, healthcare and behavioural factors influenced the incidence and prevalence of COVID-19 in other parts of the world,^3,4,5,6,7,8,9,10,11,16^ there is a knowledge deficit regarding what contributed to the high incidence in Gauteng, one of the hardest hit provinces in South Africa. Moreover, no study has examined these factors from the perspective of senior healthcare managers, who played a critical role in managing the pandemic. Understanding their experiences is crucial for informed decision-making to address any potential resurgence of COVID-19 and the spread of future infectious diseases. Therefore, this study aimed to explore the perceptions and experiences of senior managers within the Gauteng Department of Health (GDoH) and their insights into the factors that contributed to the high incidence of COVID-19 in Gauteng.

## Research methods and design

### Study design

The study employed an exploratory qualitative approach to investigate the phenomenon under study.^18^ The study was conducted between 25 October and 31 October 2022.

### Setting

Gauteng is the most populous province in the country, with over 16 million residents, accounting for 26.6% of the national population.^19^ Gauteng comprises five districts, three of which are metropolitan municipalities: Johannesburg (the economic capital of South Africa), Tshwane (the administrative hub) and Ekurhuleni (home to OR Tambo International Airport, a key gateway to the Southern African Development Community [SADC]). The remaining two districts, Sedibeng and West Rand, are non-metropolitan. The GDoH supervises these five health districts, including 493 fixed and mobile public healthcare facilities. Because of the province’s high COVID-19 incidence, Gauteng was labelled the ‘epicentre’ of the pandemic in South Africa.

### Participants and sampling

The study population consisted of senior public healthcare managers from the GDoH and its five districts. The 13 participants included eight males and five females, with professional experience ranging up to 30 years. Their expertise included nursing, medicine, maternal and child health, TB, HIV and AIDS, immunisation programme coordination, communication, forensic investigation and infectious disease management, among others. Participants were purposively selected because of their direct involvement as frontline workers in combating the COVID-19 pandemic in Gauteng.

### Data collection

Once-off in-depth interviews were conducted in English with GDoH senior managers to understand their experiences regarding the factors contributing to the high incidence of COVID-19 in Gauteng province. Participants were provided with an information sheet and interview guide prior to enrollment and were again briefed at the start of the interview sessions. Because of COVID-19 protocols, interviews were conducted online via Microsoft Teams (MS Teams) to comply with social distancing. As part of a larger study, the research questions relevant to this study were: (1) How severe was the COVID-19 pandemic in Gauteng province; and (2) What factors contributed to the severity of the COVID-19 pandemic in Gauteng province? Follow-ups were made to the questions through probing. The average interview duration was 45 min. About seven potential participants failed to participate in the study because of time constraints, but this did not affect the analysis and findings as saturation was reached by the 13th interviewee (*N* = 13). Data were collected by the first author, a male Master of Public Health (MPH) student who had no prior relationship with the participants. The interview transcripts were securely stored in a password-protected Google Drive folder, accessible only to the authors.

### Data analysis

The recorded interviews were transcribed verbatim in Microsoft Word and then uploaded into NVivo 10 software for analysis. Codes were deliberated and agreed on by the authors and were linked to the text and analysed thematically. Later, the data were explored inductively to identify unanticipated codes and emerging themes, as well as any contradictions, for triangulation purposes. To safeguard participants’ confidentiality, transcripts were de-indentified and pseudonymised as Participant 1, Participant 2 and so on. Results were reported in accordance with the 32-item checklist of the Consolidated Criteria for Reporting Qualitative Research (COREQ).^20^ While all authors agreed on the initial coding framework, the first author led the analyses. The second author, an academic with a PhD and extensive qualitative research expertise, as well as the third author, an Associate Professor, reviewed the analysis for accuracy and consistency.

### Ethical considerations

This study is one component of a larger project that used a sequential mixed methods approach to investigate the impact of the COVID-19 pandemic on essential public healthcare services in Gauteng province, South Africa.^11,21^ Other study components include: drivers of COVID-19 infections in Gauteng province (the current study) and the response, challenges and lessons learned from COVID-19 against future outbreaks (study under review). The study was approved by the University of the Witwatersrand, Johannesburg’s Human Research Ethics Committee (Wits HREC) Ref No: M220149 and the National Health Research Department Ref No: NHRD-GP_202203-031. All the participants provided written informed consent before their enrollment.

## Results

The findings are summarised in two main themes, namely: Theme 1, the perceived severity of COVID-19 in Gauteng province; and Theme 2, the perceived reasons for the high incidence of COVID-19 in Gauteng. Theme 1 had no sub-themes. Theme 2 presented six sub-themes, as summarised in Table 1.

**TABLE 1:** Theme 2 with its sub-themes of senior healthcare managers’ perception of the reasons for the high incidence of COVID-19 in Gauteng province.

Theme 2: Perceived reasons for the high incidence of COVID-19 in Gauteng	
Sub-themes	Items within sub-themes
Demographic factors	Population density
Economic factors	Employment prospects and COVID-19 incidence
	Informal settlements and COVID-19 transmission
Environmental factors	Perceived impact of OR Tambo International Airport on COVID-19 incidence
Healthcare and behavioural factors	Advanced healthcare facilities and patient influx (seeking superior healthcare)
	Impact of COVID-19’s protocol non-adherence
	Misinterpretation of COVID-19 surveillance (reporting) in Gauteng
Governance factors	Corruption and embezzlement of COVID-19 funds
Sociocultural factors	Vaccine hesitancy
	Social media and COVID-19 misinformation
	Lack of COVID-19 information and treatment

COVID-19, Coronavirus disease 2019.

Although the major and most comprehensive quotations are presented alongside the theme and sub-themes in the results section (one quote per theme or sub-theme), other important quotes have been presented as Online Appendix 1 to this article.

### Theme 1: The perceived severity of COVID-19 in Gauteng province

All the study participants recounted that the burden of COVID-19 was highest in Gauteng province, compared to the other eight South African provinces. Insights into the comparison were on two fronts: infection and case fatality rates. The narratives indicated that Gauteng had the highest number of COVID-19 infections and the second highest COVID-19 deaths, after the Western Cape province, making it seem like the province was the most severely impacted by the pandemic:

‘Well, one will say it [COVID-19] was severe in Gauteng for the simple reason that we were a leading province in terms of the number of cases [*new infections*] and deaths. The cases were always high in Gauteng. Even at the height of the different waves, Gauteng was a major province.’ (Participant 5)

In terms of COVID-19 mortality, the Western Cape province was reported to have recorded the highest number of COVID-19 deaths, followed by Gauteng province. One participant extrapolated figures from the COVID-19 National Surveillance System to justify the claims:

‘Also, when you compare the deaths, the Western Cape has the highest number of deaths sitting at about 22,000. Followed by us (Gauteng), I think we’ve got about 21,000 deaths.’ (Participant 1)

Some participants raised unanswered questions at the time this study was conducted, requesting evidence-based information about the reasons for the high burden of COVID-19 in Gauteng compared to other provinces:

‘The death rates were higher in Gauteng compared to Limpopo, I don’t know why it was like that. Maybe research is needed to say why the impact of COVID-19 in the rural areas was not as severe as compared to urban provinces.’ (Participant 10)

### Theme 2: Perceived reasons for the high incidence of COVID-19 in Gauteng province

The reasons for the high incidence of COVID-19 in Gauteng province were grouped into demographic, economic, sociocultural, environmental, health-seeking behaviour and governance factors (six sub-themes).

#### Sub-theme 2.1: Demographic factors

**Population density:** According to most participants, the high population density in Gauteng province and its metropolitan cities was the major factor that contributed to the high prevalence of COVID-19 in Gauteng. The effect of the population density led to a fast spread rate of COVID-19 infections and the deaths that emanated thereof:

‘If you recall, there is public knowledge that the impact [*of COVID-19*] and the peaks were higher in Gauteng than in other provinces because Gauteng is a small province with a high population.’ (Participant 4)

However, there were three diverse opinions regarding the geographical variations of COVID-19 infection rates among the five districts in Gauteng province. The first group of participants were the majority who held the perception that the burden of the pandemic was heavier in the three most densely populated metropolitan districts compared to the two non-metropolitan districts:

‘The most affected district was the Johannesburg metro [*metropolitan*], being a highly dense geographical area. Then the least affected were the smaller districts like West Rand and Sedibeng which are less populated. So basically, the most affected were Johannesburg, Tshwane, and Ekurhuleni which are the metros where most of our people stay and are densely populated.’ (Participant 9)

The second group of participants were few and held the view that although there was an uneven distribution of COVID-19 cases in Gauteng, it was not necessarily the most populated area that had the most cases. According to this group and contrary to public knowledge, some less dense localities within the province experienced higher COVID-19 infections and/or incidence:

‘It may shock you that the less populated districts of Sedibeng and West Rand may have more rates of [*COVID-19*] infections than the highly populated districts.’ (Participant 8)

Lastly, the third category was made up of one isolated participant who argued that the impact of COVID-19 was the same among the five districts in Gauteng province irrespective of whether a particular district was a metropolitan district or not:

‘I think all districts within the [*Gauteng*] province were affected the same, I wouldn’t say there was one district that was affected more than the others. Within Gauteng, districts are more or less the same sizes so you wouldn’t have such an effect.’ (Participant 12)

Overall, the participants seem to suggest that population density correlated with COVID-19 incidence at the Gauteng provincial level but at the district level, a minor fraction of the participants believed that this was not necessarily the case.

#### Sub-theme 2.2: Economic factors

**Employment prospects and COVID-19 incidence:** The Gauteng province is a host to mines and major commercial cities of South Africa such as Johannesburg and Pretoria, which are highly industrialised and urbanised compared to other provincial cities. As such, the participants’ experiences show that many unemployed people are always attracted to Gauteng, which led to population concentration and consequently eased the spread of COVID-19 infections:

‘People come from other provinces and countries to Gauteng for job proposals, and they are working here, but they originate from other provinces. Some who are not working come here to look for jobs. So that is why we were so affected [*by COVID-19*].’ (Participant 1)

**Informal settlements and COVID-19 transmission:** The existence of informal settlements in Gauteng province that are predominantly highly dense seemed to have exacerbated COVID-19 infections. Informal dwellings are often attributed to poverty and in this context are a habitat for poor miners, showing that socioeconomic factors can facilitate the spread of infectious diseases. One such well-known informal settlements in South Africa is the Alexandra township in Johannesburg, Gauteng province:

‘Alexandra and areas where there were more mining or informal settlements had more [*COVID-19*] infections.’ (Participant 7)

#### Sub-theme 2.3: Environmental factors

**Perceived impact of OR Tambo International Airport on COVID-19 incidence:** According to many participants, the OR Tambo International Airport, which is the main air hub of entry and exit to or from the entire Southern Africa, located in the Ekurhuleni district in Gauteng province, was one major factor that contributed to increasing COVID-19 incidence in Gauteng. It attracted a high influx of international and local travellers who were tested and if found infected were quarantined and reported as Gauteng COVID-19 cases. In effect, these COVID-19 cases were transported into Gauteng but had to be registered as an incidence in the province:

‘People from various international places congregating in Gauteng come through the OR. Tambo airport. That was the busiest place where the danger [*COVID-19*] came from because people came from various international destinations to Gauteng through the OR Tambo airport.’ (Participant 13)

#### Sub-theme 2.4: Healthcare and behavioural factors

From the experiences of senior managers, several health factors pulled a mass population into Gauteng, which served as a breeding ground and facilitated the spread of COVID-19 in the province. These factors and their impact included:

**Advanced healthcare facilities and patient influx (seeking superior healthcare):** Most of the participants stated that Gauteng province is home to the best tertiary, central, referral and academic hospitals in South Africa and the entire SADC region. The argument was that these improved healthcare facilities, infrastructure and services offer specialised care. Consequently, these advanced services are health-pulling factors for people from underserved provinces and neighbouring countries seeking healthcare in Gauteng province. This resulted in congestion as it was the case, leaving room for easy transmission of the coronavirus, hence, a high incidence of the pandemic in the province. In the words of some participants:

‘[*W]*hen people feel sick [*of COVID-19, for example*] they come to Gauteng to seek medical treatment because we have some specialized and tertiary hospitals, and maybe they believe that our hospitals are good.’ (Participant 3)

**Impact of COVID-19 protocol non-adherence:** The non-adherence to the COVID-19 protocol was reported to have contributed to the incidence of COVID-19 vis-à-vis the high population in Gauteng compared to other less populated provinces. In other words, poor adherence to COVID-19 regulations was common in all provinces, but it particularly affected Gauteng province because this opened the doors of fast contamination because of the population concentration. The wearing of face masks and social distancing were the two main non-pharmaceutical interventions highlighted from the WHO guidelines against COVID-19. Hence, one participant explained as follows:

‘I regret that we have had more [*COVID-19*] infections and deaths than we should have had due to compliance, especially to the non-pharmaceutical measures that were less than ideal. We had people not wearing the masks the way they should and others ignoring the social distancing.’ (Participant 4)

**Misinterpretation of COVID-19 surveillance (reported cases) in Gauteng:** A few participants argued that the perception that Gauteng had the highest incidence of COVID-19 in the country was misleading because the province had a better surveillance system than other provinces. Hence, Gauteng effectively screened, tested and reported more cases than other provinces in the country, which lacked adequate surveillance of the pandemic:

‘The capacity of the health system to report was beneficial. We were able to report and quantify the burden [*of COVID-19*], unlike in other provinces where they may not necessarily have had as much capacity or as much reporting ability as well. I think, as Gauteng, we were able to quantify it quite reasonably well.’ (Participant 3)

#### Sub-theme 2.5: Governance factors

**Corruption and embezzlement of COVID-19 funds:** According to some participants, corruption and financial malpractice in COVID-19 PPE funds in the GDoH posed challenges in Gauteng’s response to the COVID-19 pandemic. These malpractices meant that healthcare workers were inadequately protected and could not perform efficiently. This enabled high transmissibility of the coronavirus in Gauteng:

‘Corruption! We are the province [*Gauteng*] that was hit by corrupt tendencies because our systems were not well tuned for the delivery of this type of unplanned service delivery. It was an emergency and people used the [*PPE*] emergency procurements to rip us off. We were in the newspapers, and it affected our performance. I think we were weak in terms of procurement which is why some people had to be corrupted.’ (Participant 10)

#### Sub-theme 2.6: Sociocultural factors

**Vaccine hesitancy:** Vaccine hesitancy is a critical factor perceived by most of the participants to have escalated the severity of COVID-19 in Gauteng. The participants added that the COVID-19 vaccine hesitancy was instigated by the propaganda of false information that circulated claiming that COVID-19 vaccines contained chips to monitor and control humanity. Furthermore, because pork was propagated as one of the vaccine’s ingredients besides other conspiracy theories, anti-pork religious communities distanced themselves from being vaccinated. Such misguided information led to vaccine hesitancy and the virus became opportunistic and spread even faster in the densely populated province:

‘In fact, we experience high numbers of deaths in Muslim communities because when the vaccines became available, there was an issue around the pork and the ingredients that were used in the vaccines.’ (Participant 11)

Although COVID-19 vaccine hesitancy was a global issue and was predominantly among the general population with less health literacy, it was paradoxically experienced among Gauteng healthcare workers, which enabled a high transmission because of the close interaction of healthcare workers with their families and clients, thereby increasing the incidence rate of COVID-19 in the province. Some participants lamented as follows:

‘[*COVID-19*] vaccine hesitancy was an issue. Even amongst the healthcare workers, the uptake was quite very low.’ (Participant 1)

On the other hand, some participants expressed frustrations and worries observing that the population had become reluctant to vaccination and booster shots, and this may sustain the high rates of COVID-19 in Gauteng province. Despite the reluctance of some people to vaccinate, the GDoH was shown to still be conducting health promotion and awareness to continue with its vaccination agenda against the pandemic:

‘[*B*]ut what we are now experiencing is the fatigue. We have people who have coverage fatigue. People are now reluctant to get the vaccine because they see that people are no longer dying. People have relaxed, that is the main challenge that we are now seeing. The energy is no longer there in the community, but overall, we are continuing with encouragement and creating demand for the vaccine.’ (Participant 5)

Consequently, COVID-19 vaccine hesitancy with implications of demand waning, coupled with storage challenges led to the expiration of some vaccines, which were disposed of at the expiration date as they became unfit for consumption. This impacted Gauteng province the most because the province was a hotspot of the pandemic, hence, an increase in COVID-19 incidence in the jurisdiction:

‘Another thing that remains a soreness in my heart is the number of vaccines that this country acquired but they remain wasted.’ (Participant 7)

**Social media and COVID-19 misinformation:** According to all the participants, social media was an adverse instrument during COVID-19. It misinformed the public by spreading false information about COVID-19, which exacerbated vaccine hesitancy. This particularly affected Gauteng as a more urbanised province with countless social media platforms and Internet access:

‘There was no buy-in because communities thought they were going to die. Social media also contributed to the communities having fears and not getting the vaccines.’ (Participant 9)

Some of the concerned participants expressed their desires for the regulation of social media to limit the infodemics as highlighted by this senior manager:

‘There should be an engagement with the corporations that are behind Facebook, YouTube, etc, to say that only registered and accredited health professionals should provide health advice. We have every Tom and Dick who goes on YouTube to say NO, take this, don’t vaccinate!’ (Participant 2)

**The lack of COVID-19 information on vaccines and treatment:** The lack of scientific information on specific treatments and vaccines for COVID-19 at the early stage of the pandemic was one enabler of COVID-19 infections in Gauteng coupled with the fast spread of the pandemic in an already vulnerable province because of its high population density and the other factors highlighted above. Some of the participants recounted the extent to which some of the ineffective measures were promoted by stakeholders as follows:

‘COVID-19 had a severe impact on us in Gauteng and Tshwane district in particular when we had limited knowledge about the virus, it affected how we managed it. We were building the aeroplane while we were already flying, we did a lot of things that were not scientifically based, I am referring to fumigation.’ (Participant 2)

**FIGURE 2:**
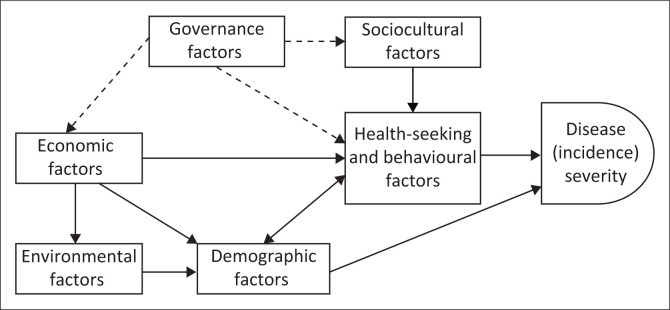
A model for investigating and responding to the human factors that enable the transmission of infectious diseases.

A critical examination of the results of this study produces a model (Figure 2) that can be used to investigate and respond to the human factors that enable the transmission of infectious diseases. The model (Figure 2) depicts three main hierarchical factors that influence the severity of an infectious disease such as COVID-19 (Ebola, cholera, etc.). Firstly, health-seeking and behavioural factors like the lack of information and treatment, accompanied by vaccine hesitancy and failure to adhere to protocols, were the factors most likely to be influenced by sociocultural factors, for example, social media infodemics and poor governance such as the embezzlement of funds allocated to treatment and PPEs. Secondly, demographic factors such as population density adversely affect the severity of infectious diseases. Several other sociocultural and environmental (e.g. airports) factors also contribute to high population density, which increases the transmission of infections and burdens health facilities, causing a surge in infections. Thirdly, economic factors, namely poverty, informal settlements, a lack of income and economic opportunities, may cause high population concentration as well as undermine health-seeking behaviours, further exacerbating the disease incidence. The dashed lines emanating from government factors in Figure 2 represent the regulatory role the government should play, which will indirectly influence all other factors. In addition to the disease severity or incidence, other outcomes that could be investigated using the model may include prevalence and mortality, or other measures as deemed fit by the researcher. The discussion section will present recommendations on how best these factors can be addressed.

## Discussion

This study explored the reasons for the high incidence of COVID-19 in Gauteng province, based on insights from GDoH senior managers. Two main themes emerged from the study. The first theme highlighted Gauteng as the province with the highest incidence of COVID-19 in South Africa. The second theme detailed key health and behavioural factors including poor protocol adherence that exacerbated the spread of COVID-19. Economic challenges such as employment prospects and informal settlements, sociocultural enablers, namely vaccine hesitancy, social media misinformation, limited vaccine and treatment options and environmental factors such as the OR Tambo International Airport contributed to high population density and heightened the infections and transmission of COVID-19, while governance issues such as corruption in PPE procurement undermined the pandemic’s response.

The surge in COVID-19 cases in South Africa was partly driven by socioeconomic challenges such as overcrowding and a lack of clean water, alongside poor adherence to isolation guidelines and high alcohol consumption.^22^ By February 2022, only 28.95% of the South African population had received vaccinations, and as of September 2024, this figure stands at 41%.^23^ Against this backdrop, the findings presented in this article gain importance, particularly as Gauteng was identified as the ‘epicentre’ of COVID-19 in South Africa, experiencing the highest incidence rates and significant mortality.^24^ The severity of infections in the province is attributed to several intertwined and multi-faceted factors that contributed to the spread of the virus as expressed in the interviews and Figure 2.

This study highlights that the most significant human factors that influenced the severity of COVID-19’s incidence in Gauteng province were health-seeking and behavioural factors. A lack of information and treatment at the early phase of the outbreak, vaccine hesitancy and misinformation and poor protocol adherence among other factors were significant barriers to vaccine uptake. Likewise, previous research identified knowledge about COVID-19 and personal values as significant determinants of whether individuals complied with health guidelines.^25,26,27^ Addressing vaccine hesitancy through comprehensive public health education is essential to foster trust and enhance adherence to health measures.

This study also demonstrates a perceived adverse association between population density and rapid COVID-19 transmission, particularly in overcrowded informal settlements with inadequate sanitation. The findings align with previous studies showing that dense populations are prone to higher COVID-19 transmission rates, especially when combined with poor living conditions, inadequate housing and healthcare infrastructure.^3,4,5^ Residents of informal settlements often experience poor living conditions, including overcrowding and limited access to clean water and sanitation, which further exacerbate their vulnerability to the virus.^29^ While population density alone was not the sole determinant of rising infection rates, it exacerbated the effects of other risk factors such as overcrowding and limited access to essential resources as found in a previous Gauteng study on the social determinants of health during COVID-19.^11^ To address the risks associated with high population density, investment in public health infrastructure is crucial. Specifically, improving sanitation and healthcare access in informal settlements will help curb any potential resurgence and the transmission of future outbreaks. As inadequate sanitation and overcrowding exacerbate vulnerability to the virus, expanding infrastructure in high-density areas can reduce the risks posed by these conditions.

Socioeconomic factors, namely unemployment and poverty, played a crucial role in shaping treatment-seeking behaviour during COVID-19, as per this study. Similarly, other studies have investigated socioeconomic inequalities in the spread of COVID-19 and have established a connection between elevated poverty rates and confirmed COVID-19 cases.^30,31^ Additionally, the relationship between education and COVID-19 incidence is complex, with conflicting evidence regarding whether lower educational attainment correlates with higher infection rates.^32,33,34^ Studies on unemployment also show mixed results; for instance, 45.8% indicated a positive correlation with infection rates, while others found no definitive link.^35,36,37^ Urban areas have played a pivotal role in the initial spread of COVID-19, with numerous studies confirming their significant contribution to transmission.^4^ Furthermore, larger household sizes are associated with an increased risk of transmission, supported by 61.8% of studies on overcrowding.^36,38^

As found in this study, other research has illustrated the influence of socioeconomic factors on COVID-19 compliance with health measures, complicating efforts to control the virus transmission.^11^ Therefore, in Gauteng, the influx of individuals seeking economic opportunities exacerbates population density in already congested areas, which will increase the risk of transmission of potential future outbreaks. Targeted public health interventions should be implemented in informal settlements, such as improved sanitation, vaccination campaigns and public health education. Enhancing policies to manage the economic impacts of migration will also help manage the strain on public health in the province and prevent future outbreaks or at least manage their transmission rates.

Similar to this study, a systematic review considered airports as an environmental factor and demonstrated similar findings of its effect in enabling the spreading of COVID-19 infections.^5^ Likewise, this study found that social media misinformed the public about COVID-19 and fuelled vaccine hesitancy. This aligns with previous studies that highlighted social media infodemics during COVID-19 but advised that health systems should equally use social media to counter health misinformation.^8,11^ These findings underscore the substantial challenges posed by socioeconomic, environmental and sociocultural factors, particularly population density, social media and overcrowding, in managing public health crises such as COVID-19. By understanding these dynamics, we can better address the factors that impact the prevalence and severity of infectious diseases.

Corruption and mismanagement in the procurement of PPE significantly hindered the effectiveness of the pandemic response in Gauteng. These issues not only left healthcare workers inadequately protected but also contributed to increased rates of virus transmission among healthcare staff and the broader community. Our findings align with existing literature indicating that low levels of trust in government institutions, including law enforcement, are associated with non-adherence to social distancing guidelines.^28^ While trust in the police is an area that has received less attention in research, it plays a role in the broader context of trust in authority. Conversely, trust in government has been well documented as a critical predictor of compliance with social distancing measures among the general population.^39^ The erosion of public trust because of multiple conflicting messages from government authorities on disease preventive measures in this study can create a significant barrier to effective public health strategies, as individuals may be less inclined to follow guidelines perceived as untrustworthy or poorly enforced. This underscores the need for transparent governance and effective communication from authorities during health crises. By addressing corruption and enhancing the management of healthcare resources, governments can foster greater public trust, which is essential for encouraging adherence to health measures. Strengthening trust not only aids in compliance but also enhances the overall effectiveness of responses to pandemics and similar public health challenges.

The above recommendations aimed at combating or curbing the transmissibility of future infectious diseases, outbreaks or pandemics, are summarised in Figure 2 (developed from the current findings). The model (Figure 2) has been presented in its simplest form, which allows clarity to simple questions: (1) what are the health and behavioural, demographic, economic, environmental, sociocultural or governance factors that enable the transmission of an infectious disease (COVID-19, cholera, ebola, etc.); and (2) how can the government, communities or public health respond to these factors? Although research may focus on any one or a few of the factors, a more comprehensive inquiry into all components of the model will yield a holistic response mechanism to immediately halt transmission. The current model (Figure 2) is unique in its simple form and can implore both qualitative and quantitative studies. It aligns with complex studies, which suggest the incorporation of socioeconomic, environmental, internal and external factors, among others, in the understanding of the factors that contribute to infectious disease transmission.^40^

## Limitations and strengths

The study is based on recounted information provided by GDoH senior managers, which may be subject to social desirability bias. In addition, the sample was limited to public healthcare managers, which may not fully represent the broader healthcare sector or other critical perspectives from the private healthcare sector. However, the participants (GDoH senior managers) played an essential role in managing the pandemic, which justifies the use of the purposive sample technique. Their field experiences and perspectives remain a major strength of the study, as they offer valuable first-hand experience in the management of the COVID-19 crisis. The alignment of these findings with existing literature supports the external validity of the study. However, caution should be exercised when attempting to generalise the findings or apply them to other contexts and settings. While the model in Figure 2 is invaluable for understanding the human factors influencing infectious disease spread or transmission, it does not account for biological or genetic factors that may affect the transmissibility of infectious diseases. Nonetheless, the model is still of great importance in the prevention of infections considering that public health’s primary objective is disease prevention and a better understanding of the human factors that spread diseases are crucial in developing solutions or interventions for preventive purposes.

## Conclusion

This study has highlighted the complex interplay of human factors that contributed to the high incidence of COVID-19 in Gauteng. The province’s high population density, economic and sociocultural challenges, environmental factors and governance issues all played critical roles in exacerbating COVID-19 infections. Effective future responses must focus on addressing these factors through targeted infrastructure improvements, better vaccine promotion, enhanced healthcare capacity and transparent governance. By adopting a comprehensive, multifaceted approach, future infectious disease outbreaks can be better managed.
